# Tylophorine, a phenanthraindolizidine alkaloid isolated from *Tylophora indica* exerts antiangiogenic and antitumor activity by targeting vascular endothelial growth factor receptor 2–mediated angiogenesis

**DOI:** 10.1186/1476-4598-12-82

**Published:** 2013-07-29

**Authors:** Sarita Saraswati, Pawan K Kanaujia, Shakti Kumar, Ranjeet Kumar, Abdulqader A Alhaider

**Affiliations:** 1 Camel Biomedical Research Unit, College of Pharmacy and Medicine, King Saud University, Riyadh, Kingdom of Saudi Arabia; 2 Department of Microbiology, South Campus, University of Delhi, New Delhi, India; 3 Bioinformatic Centre, North-Eastern Hill University, 793022 Shillong, India; 4 Laser Applications and Holography Laboratory, Instrument Design Development Centre, Indian Institute of Technology Delhi, 110016 New Delhi, India; 5 Department of Physiology, College of Medicine, King Saud University, Riyadh, Saudi Arabia

**Keywords:** Tylophorine, VEGFR2, Antiangiogenesis, Microvessel density, Molecular docking

## Abstract

**Background:**

Anti-angiogenesis targeting VEGFR2 has been considered as an important strategy for cancer therapy. Tylophorine is known to possess anti-inflammatory and antitumor activity, but its roles in tumor angiogenesis, the key step involved in tumor growth and metastasis, and the involved molecular mechanism is still unknown. Therefore, we examined its anti-angiogenic effects and mechanisms *in vitro* and *in vivo.*

**Methods:**

We used tylophorine and analyzed its inhibitory effects on human umbilical vein endothelial cells (HUVEC) in vitro and Ehrlich ascites carcinoma (EAC) tumor *in vivo*.

**Results:**

Tylophorine significantly inhibited a series of VEGF-induced angiogenesis processes including proliferation, migration, and tube formation of endothelial cells. Besides, it directly inhibited VEGFR2 tyrosine kinase activity and its downstream signaling pathways including Akt, Erk and ROS in endothelial cells. Using HUVECs we demonstrated that tylophorine inhibited VEGF-stimulated inflammatory responses including IL-6, IL-8, TNF-α, IFN-γ, MMP-2 and NO secretion. Tylophorine significantly inhibited neovascularization in sponge implant angiogenesis assay and also inhibited tumor angiogenesis and tumor growth *in vivo*. Molecular docking simulation indicated that tylophorine could form hydrogen bonds and aromatic interactions within the ATP-binding region of the VEGFR2 kinase unit.

**Conclusion:**

Tylophorine exerts anti-angiogenesis effects via VEGFR2 signaling pathway thus, may be a viable drug candidate in anti-angiogenesis and anti-cancer therapies.

## Background

Angiogenesis, the formation of new blood vessels by sprouting from pre-existing endothelium [1], one of the characteristic of malignant neoplasia development [2]. Angiogenesis blockade has been shown to be an effective strategy in inhibiting tumor growth and metastasis [3]. A major pro-angiogenic cytokine is vascular endothelial growth factor (VEGF) which comprises several isotypes, including VEGF-A (vascular permeability factor), VEGF-B, VEGF-C and VEGF-D, as numerous splice variant isoforms [4]. VEGF exerts its biological actions on the endothelial cells is mediated by two types of receptor tyrosine kinases (RTKs), namely VEGFR1 (Flt-1) and VEGFR2 (KDR/ Flk-1) with high affinities [4]. VEGFR2 plays an important role in mediating the mitogenesis and permeability of endothelial cells. Autophosphorylation of Tyr^1175^ on VEGFR2 is crucial for endothelial cell proliferation, and leads to the activation of downstream signaling events including Src family kinase [5], focal adhesion kinase (FAK) [6,7], phosphoinisitide 3 kinase/AKT kinase, Mammalian target of rapamycin (mTOR) [8], protein kinase C/protein kinased D, mitogen extracelluar kinase/ extracellular signal related kinase (ERK) that subsequently promote proliferation, migration, and tube formation of endothelial cells in pre-existing vasculature. Recently many studies showed the important role of VEGFR2 in potential drug discovery and molecular mechanism research [9]. Considering anti-angiogenesis therapy is to target endothelial cells that support tumor growth rather than cancer cells themselves, VEGFR2 has become an important therapeutic target for cancer anti-angiogenesis therapy [10-13].

The National Institutes of Health (NIH) website provides a basic summary of anti-angiogenic drugs that were or are still currently under clinical investigations (http://www.cancer.gov/clinicaltrials/developments/antiangio-table). These include monoclonal antibodies targeting VEGF ligands or VEGFRs [14], soluble receptors that sequester ligands [15] and small molecule inhibitors that inhibit kinase activity [16]. Three drugs developed for their anti-angiogenic actions, bevacizumab (Avastin®), sunitinib malate (Sutent®, SU11248) and sorafenib (Nexavar®, BAY 43-9006), have been approved by the United States Food and Drug Administration for treatment of patients with specific types of cancer—all three inhibit VEGF signaling by blocking VEGF ligand or VEGFR [17]. However, serious side effects, such as hypertension, bleeding and gastrointestinal perforation, have been associated with currently available anti-VEGF agents, limiting their chronic use [17]. Hence, there is an urgent need to find a molecule that can be more specific and less toxic for the treatment of cancer, particularly agents that exhibit activity against drug-resistant strains, completely sterilize the infection, or shorten the duration of drug therapy and thus promote drug compliance.

**Figure 1:**
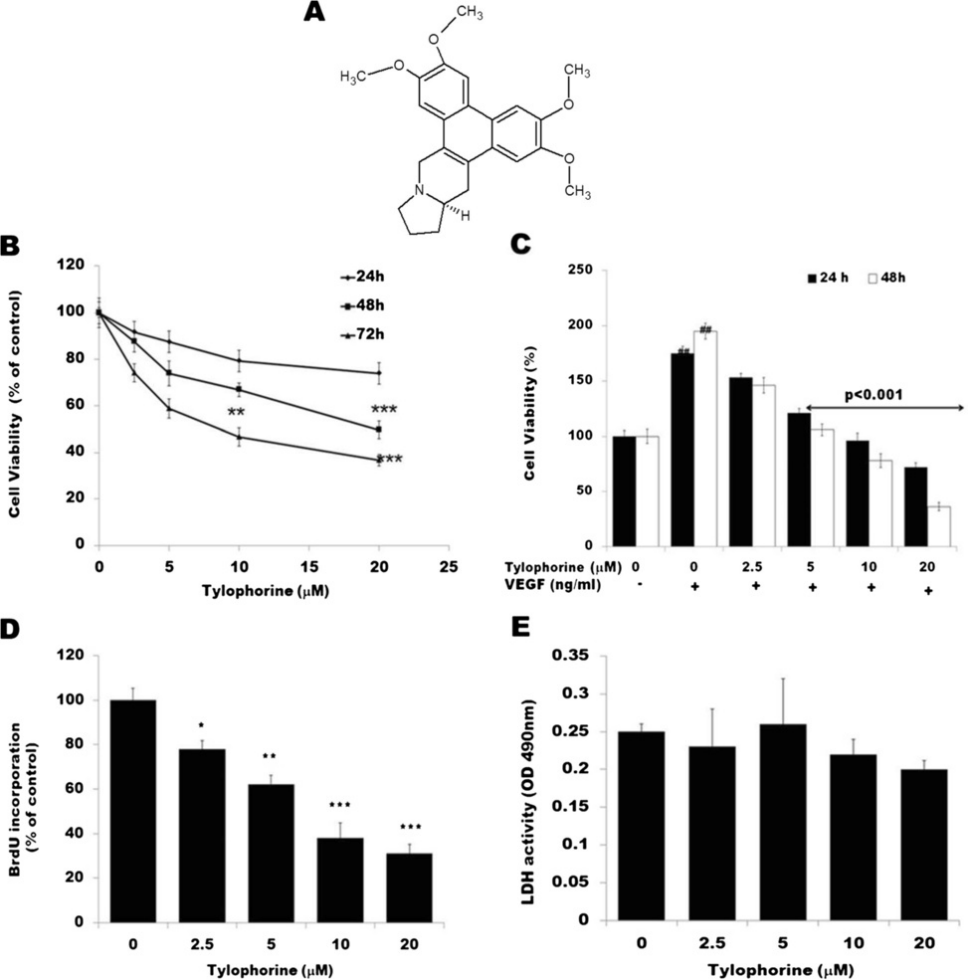
**Effect of tylophorine on cell proliferation in HUVECs. (A)** Chemical structure **(B)** Under normal culture condition. HUVECs were cultured in ECGM containing 20% FBS, then cells (5 × 10^4^ cells/well) were treated with DMSO (0.1%) or various concentrations of tylophorine for 24, 48 and 72 h. Cell viability was determined by MTT assay. Cells receiving only DMSO (0.1%) served as a vehicle control. Data were expressed as percentages of the vehicle control (100%) as mean ± SEM, n = 6 wells. **p < 0.01; ***p < 0.001 versus control group. **(C)** Under VEGF-stimulated condition HUVECs (5 × 10^4^ cells/well) were starved with ECGM supplemented with 0.5% FBS for 24 h, and then treated with or without VEGF (10 ng/mL) and DMSO (0.1%) or various concentrations of tylophorine for another 24 and 48 h. Data were expressed as percentages of the vehicle control (100%) as mean ± SEM, n = 6 wells. **(D)** Effects of tylophorine on DNA synthesis was examined by BrdU cell proliferation enzyme linked immunosorbent assay. Data were expressed as percentages of the vehicle control (100%) as mean ± SEM, n = 6 wells. *p < 0.05; **p < 0.01; ***p < 0.001 versus control group. **(E)** Tylophorine administration did not result in LDH release from endothelial cells as studied with LDH cytotoxicity assay kit indicating that tylophorine posed little cytotoxicity effects upon HUVECs. Data were expressed as percentages of the vehicle control (100%) as mean ± SEM, n = 6 wells.

Tylophorine (Figure 1A) and its analogs are phenanthroindolizidine alkaloids, isolated from *Tylophora indica* (Asclepiadaceae) [18]. *Tylophora indica* has been included as an official drug in the Bengal pharmacoepia of 1884 [19]. The leaves of this plant have been used for the treatment of asthma as well as bronchitis, rheumatism and dysentery in India. These alkaloids possesses anticancer [20-24], anti-inflammatory [19,25], anti-ameobicidal [26] and anti-viral [27] activity. Several key metabolic enzymes, including thymidylate synthase [28] and dihydrofolate reductase have been reported as biological targets of tylophorine alkaloids [29]. Tylophorine derivatives also inhibits activator protein-1–mediated, CRE-mediated, and nuclear factor kappaB (NF-κB)-mediated transcription [30,31]. Tylophorine arrests the cells at G1 phase in HepG2, HONE-1, and NUGC-3 carcinoma cells and down regulates cyclin A2 expression [32]. Preliminary studies illustrate the potential of tylophorine as a new class of anticancer drugs. However, the molecular mechanism responsible of its inhibitory effects on cancer cell growth is largely unknown. In this study, we evaluated for the first time how tylophorine inhibits tumor angiogenesis by targeting key signaling pathways on human endothelial cells and *in vivo* mouse model. Our results demonstrate that tylophorine significantly inhibited VEGF-stimulated endothelial cell proliferation, migration and tube formation *in vitro*. Tylophorine inhibited neovascularization in sponge implant angiogenesis assay *in vivo* and further attenuated tumor associated angiogenesis. Furthermore, mechanistically, tylophorine suppressed VEGFR2-mediated signaling pathway. Meanwhile, the structure-based interaction between tylophorine and VEGFR2 was found to be stable conformation based on *in-silico* analysis which revealed that hydrogen bond and aromatic interactions were formed. Taken together our results suggest that tylophorine could be used as a potential anti-angiogenesis agent that targets VEGF/VEGFR2 signaling pathways and inhibits tumor induced angiogenesis.

## Results

### Tylophorine inhibited cell viability in endothelial cells

Angiogenesis is primarily initiated by growth factors therefore we tested whether tylophorine decreases VEGF-mediated HUVEC viability and proliferation. We found that when HUVECs were cultured in normal cell culture medium (ECGM supplemented with 20% FBS) in absence of VEGF, tylophorine inhibited cell viability in a dose- and time-dependent manner. Significant cell viability inhibitory effect of tylophorine was observed in HUVECs at concentrations more than 10 μM (Figure 1B). As shown in Figure 1C, the proliferation of endothelial cells stimulated by VEGF was markedly decreased after tylophorine treatment ranging from 2.5 to 20 μM at different time intervals of 24 and 48 h indicating extracellular VEGF acted as a strong attractant for endothelial cells proliferation. Tylophorine alone inhibited the growth of HUVEC in dose dependent manner (Additional file 1: Figure S1A). As detected by BrdU incorporation assay (Figure 1D), DNA synthesis of HUVECs was also significantly inhibited by tylophorine in a dose-dependent manner. To further examine whether tylophorine would result in toxic effects of HUVEC, LDH cytotoxic assay was carried out. As shown in Figure 1E, Tylophorine caused minute toxicity on HUVECs.

### Tylophorine inhibited VEGF-induced endothelial cell migration and invasion and tube formation of HUVECs

**Figure 2:**
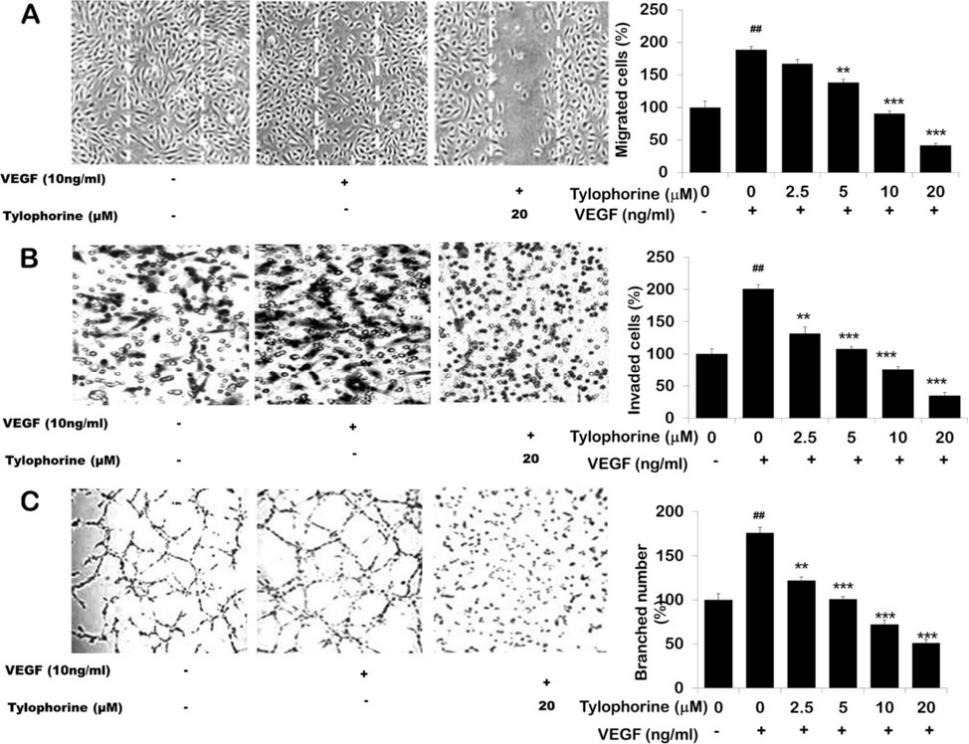
**Tylophorine inhibited VEGF-induced endothelial cell migration and invasion and tube formation of HUVECs. (A)** Effect of tylophorine on VEGF-induced cell motility (wound-healing assay). Confluent HUVEC monolayers on 0.1% gelatin-coated six-well plates were scratch wounded. The cells were treated with various concentrations of tylophorine with 0.5% FBS and 10 ng/mL VEGF for 16 h. Representative fields were photographed, × 100 magnification. Graph shows the quantitative effect of tylophorine on VEGF-induced HUVEC motility. Data were presented as mean ± SEM, n = 6 wells. ^##^p < 0.01 VEGF-treated group versus no VEGF-treated group; **p < 0.01; ***p < 0.001 versus VEGF-stimulated group. **(B)** Effect of tylophorine on VEGF-induced invasion of HUVEC through Matrigel in 24 h. Representative fields were photographed, × 100 magnification. Graph shows the quantitative effect of tylophorine on VEGF-induced HUVEC invasion. Data were presented as means ± SEM, n = 6 wells. ^##^ p < 0.01 VEGF-treated group versus no VEGF-treated group; **p < 0.01; ***p < 0.001 compared with VEGF-stimulated group. **(C)** Effect of tylophorine on VEGF-induced capillary-like tube formation of HUVEC through Matrigel in 24 h. Representative fields were photographed, × 100 magnification. Graph shows the quantitative effect of tylophorine on VEGF-induced HUVEC tube formation. Data were presented as mean ± SEM, n = 6 wells. ^##^p < 0.01 VEGF-treated group versus no VEGF-treated group; **p < 0.01; ***p < 0.001 versus VEGF-stimulated group.

Cell migration is an essential step in angiogenesis [33]; therefore we investigated the effects of tylophorine on the chemotactic motility of the endothelial cells by using wound-healing (Figure 2A) assay. The results showed that tylophorine significantly inhibited VEGF-induced HUVECs migration in a dose-dependent manner ranging from 2.5 μM to 20 μM. Directional motility and matrix degradation are crucial for angiogenesis sprouting therefore, we next examined the effect of tylophorine on the invasion ability of HUVECs using the Boyden chamber assay. As shown in Figure 2B, a large number of cells migrated to the lower side of membrane in the transwell chamber after stimulation with VEGF. However, the number of invaded cells were significantly low in the presence of tylophorine (p < 0.001). The maturation of migrated endothelial cells into a capillary tube is a critical step during angiogenesis [34]. Thus, we investigated its effect on HUVEC tube formation. When HUVECs were seeded on the growth factor–reduced matrigel, robust tubular-like structures were formed in the presence of VEGF (Figure 2C). Almost 80% destruction of tube network was observed when HUVECs were incubated with tylophorine at 10 μM (p < 0.001). Taken together, tylophorine suppressed VEGF-induced angiogenesis *in vitro* by inhibiting the migration, invasion and tubular structure formation of endothelial cells.

### Differential effect of tylophorine on the binding of VEGF to its receptors

**Figure 3:**
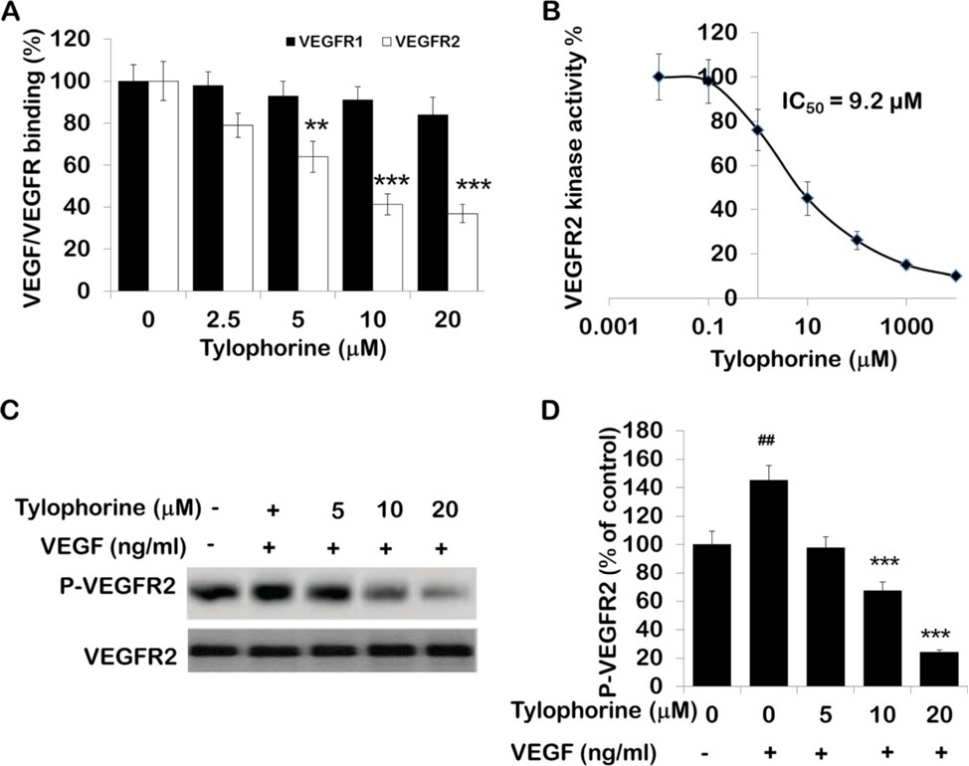
**Tylophorine inhibits VEGFR2 binding with VEGF and attenuated VEGFR2 tyrosine kinase activity. (A)** Effect of tylophorine on the binding of VEGFR1 (Flt-1) and VEGFR2 (KDR/Flk-1) to immobilized VEGF. Data were presented as means ± SEM, n = 6. **p < 0.01; ***p < 0.001 compared with control. **(B)** Inhibition of VEGFR2 kinase activity by tylophorine was analyzed using an *in vitro* HTScan® VEGF receptor 2 kinase kit (Cell Signaling Technology, Danvers, MA, USA) combined with colorimetric ELISA detection according to the manufacturer’s instructions. The reaction processed with DMSO (0.1%) served as a vehicle control. Data were expressed as percentages of the vehicle control. **(C)** Western blot analyses of effect of tylophorine on phosphorylation of VEGFR2. HUVECs were pre-treated with tylophorine followed by the stimulation with 50 ng/mL of VEGF for 2 min. Data were presented as means ± SEM, n = 6. **(D)** Quantitative densitometry of VEGFR2 phosphorylation is shown as percentage (%) of control. Data were presented as means ± SEM, n = 6. ^##^ P < 0.01 VEGF-treated group versus no VEGF-treated group; **p < 0.01; ***p < 0.001 versus VEGF-stimulated group.

Further, we investigated whether tylophorine inhibits the binding of VEGF to its receptors, VEGFR1 (Flt-1) and VEGFR2 (Flk-1/KDR). As shown in Figure 3A, tylophorine decreased the binding of VEGFR2 to immobilized VEGF with IC_50_ of ~ 12.29 μM. However, tylophorine did affected the binding between VEGF and VEGFR1 (Figure 3A) but it did not reached to significant level. Antihuman VEGFR1 antibody (AF321, R&D Systems) and antihuman VEGFR2 antibody (MAB3572, R&D Systems) were used as positive control for VEGFR1 and VEGFR2 respectively (data not shown).

### Tylophorine attenuated VEGFR2 tyrosine kinase activity

Previous studies suggested that blockage of VEGFR-2 activity could significantly limit tumoral neovascularization process [12]. Therefore, we first investigated whether tylophorine decreased P-VEGFR2 levels by inhibiting the kinase activity of VEGFR2 using an ELISA-based tyrosine kinase assay. Tylophorine was found to inhibit kinase activity of VEGFR2 (Figure 3B) with an IC_50_ of ~ 9.2 μM. SU5416, a known inhibitor of VEGFR2, was used as a positive control and showed inhibition of kinase activity with an IC_50_ of 1.5 μM (data not shown), as described previously [35].

We further examined the effects of tylophorine on phosphorylation of VEGFR2 to determine its inhibitory effect on VEGFR2-mediated signaling pathways in endothelial cells. We found that VEGFR2 was phosphorylated by the addition of exogenous VEGF to HUVECs (Figure 3C). Pretreatment of cells with tylophorine significantly blocked VEGF-induced phosphorylation of VEGFR2, without affecting overall VEGFR2 expression levels. Quantitative densitometry of protein phosphorylation is shown as percentage (%) of vehicle control (Figure 3D). The protein levels were normalized to β-actin. In addition, previous studies supported that phosphorylation of VEGFR2 could subsequently trigger multiple downstream signals that induced proliferation and differentiation activities of endothelial cells [36,37].

### Tylophorine inhibited the activation of VEGFR2-mediated signaling pathways

**Figure 4:**
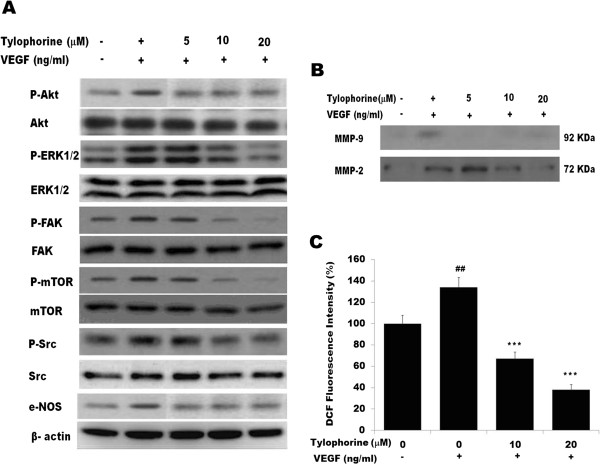
**Western blot analysis of the effect of tylophorine on VEGFR2-mediated downstream signaling. (A)** HUVECs were pre-treated with tylophorine followed by the stimulation with 50 ng/mL of VEGF for 20 min. Data were presented as means ± SEM, n = 6. **(B)**. Effect of tylophorine on VEGF-induced MMP-2 secretion from HUVECs after 20 h examined by zymography. Data were presented as means ± SEM, n = 6. **(C)**. Effect of tylophorine on HUVECs intracellular ROS level as detected by DCFH-DA staining assay. Data were presented as means ± SEM, n = 6. ^##^p < 0.01 VEGF-treated group versus no VEGF-treated group; **p < 0.01; ***p < 0.001 versus VEGF-stimulated group.

Binding of VEGFR2 with VEGF led to the activation of various downstream signaling molecules responsible for endothelial cell migration, proliferation and survival [35]. To further delineate the mechanism that underlies the anti-angiogenic effects of tylophorine, we screened some key kinases involved in VEGFR2-mediated signaling pathway. VEGF induces survival of endothelial cells (ECs) mainly via the activation of AKT [37], whereas activation of ERK1/2 MAPKs is thought to be essential for VEGF-induced proliferation [38]. To assess the effect of tylophorine on these pathways, serum-starved HUVECs were treated with VEGF for 20 minutes in the presence or absence of tylophorine and cell lysates were subjected to immunodetection using antibodies against either P-AKT (Ser^473^) or P-ERK1/2. The result showed that P-ERK1/2 is enhanced by VEGF treatment while the expression level of ERK1/2 remains unchanged. Tylophorine was found to inhibit the phosphorylation of ERK1/2 at the concentration of 20 μM without affecting total ERK1/2 expression level (Figure 4A). A recent study suggests that the AKT/mTOR pathways and Hsp90, which are critical for angiogenesis, are phosphorylated or activated by VEGFR2 activation in the endothelial cells [39]. As shown in Figure 4A, expression levels of P-AKT and p-mTOR increases by VEGF treatment. Pretreatment of the HUVECs with tylophorine significantly inhibited the phosphorylation of AKT and mTOR, while the total amount of AKT and mTOR remains unchanged. Further, the action of tylophorine on the phosphorylation of FAK and Src were determined. The result showed that tylophorine inhibited VEGF-induced phosphorylation of FAK at the dose of 10 and 20 μM and Src at the concentration of 20 μM respectively (Figure 4A). Tylophorine could evidently inhibit VEGF-stimulated eNOS expression. In addition, both the MMP-9 and MMP-2 activities were suppressed with tylophorine treatment (Figure 4B). ROS is known to be downstream signaling after VEGFR2 activation [40], therefore, we detected the ROS levels by DCFH-DA probe. The results showed that the intracellular ROS levels were significantly reduced after tylophorine administration (Figure 4C). Taken together, our result revealed that tylophorine inhibited *in vitro* angiogenesis by directly targeting VEGFR2 on the surface of endothelial cells, and further downregulating VEGFR2-mediated signaling pathway.

### Tylophorine inhibited VEGF-induced IL-6, IL-8, TNF-α, IFN-γ, MMP-2 and NO

**Figure 5:**
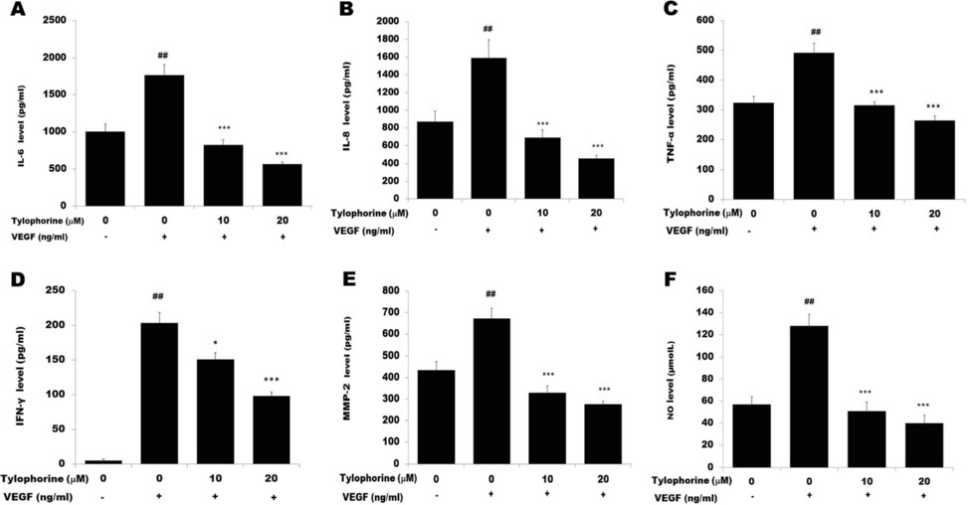
**Effect of tylophorine on secretion of VEGF-induced cytokines. (A)** IL-6, **(B)** IL-8, **(C)** TNF-α **(D)** IFN-γ **(E)** MMP-2 and **(F)** NO. Serum-starved HUVECs were preincubated with DMSO (0.1%) or tylophorine (20μM), before stimulation with VEGF (10 ng/mL), and supernatants were harvested after 24 h for cytokine assays. IL-6, IL-8, TNF-α, IFN-γ and MMP-2 were measured by sandwich ELISA following the manufacturer’s instructions (R and D systems, USA). Absorbance was determined using a microplate reader (Biorad, USA) at 450 nm. The NO levels in the HUVECs were measured with Nitric oxide colorimetric assay kit (Biovision, USA) following the manufacturer’s instructions. Absorbance was determined using a microplate reader (Biorad, USA) at 540 nm. Data were presented as means ± SEM, n = 3. ^##^p < 0.01 VEGF-treated group versus no VEGF-treated group; **p < 0.01; ***p < 0.001 versus VEGF-stimulated group.

During inflammation VEGFR activation is linked to cytokine release, pro-inflammatory molecules and leukocyte endothelial interactions, which exacerbate the inflammatory response [41]. Therefore, we investigated the effect tylophorine on endothelial cell cytokine release. As shown in Figure 5, HUVECs treated for 24 h with VEGF up-regulated the secretion of IL-6 (Figure 5A), IL-8 (Figure 5B), TNF-α (Figure 5C), IFN-γ (Figure 5D) and MMP-2 (Figure 5E). HUVECs pretreated with tylophorine, before the addition of VEGF (10 ng/mL), significantly (P < 0.001) decreased the cytokine secretion IL-6, IL-8, TNF-α, IFN-γ and MMP-2 in a dose-dependent manner (Figure 5). Further tylophorine significantly inhibited NO levels (Figure 5F, P < 0.001) in HUVEC at 24 h incubation in a dose-dependent manner.

### Tylophorine inhibited neovascularization in vivo

**Figure 6:**
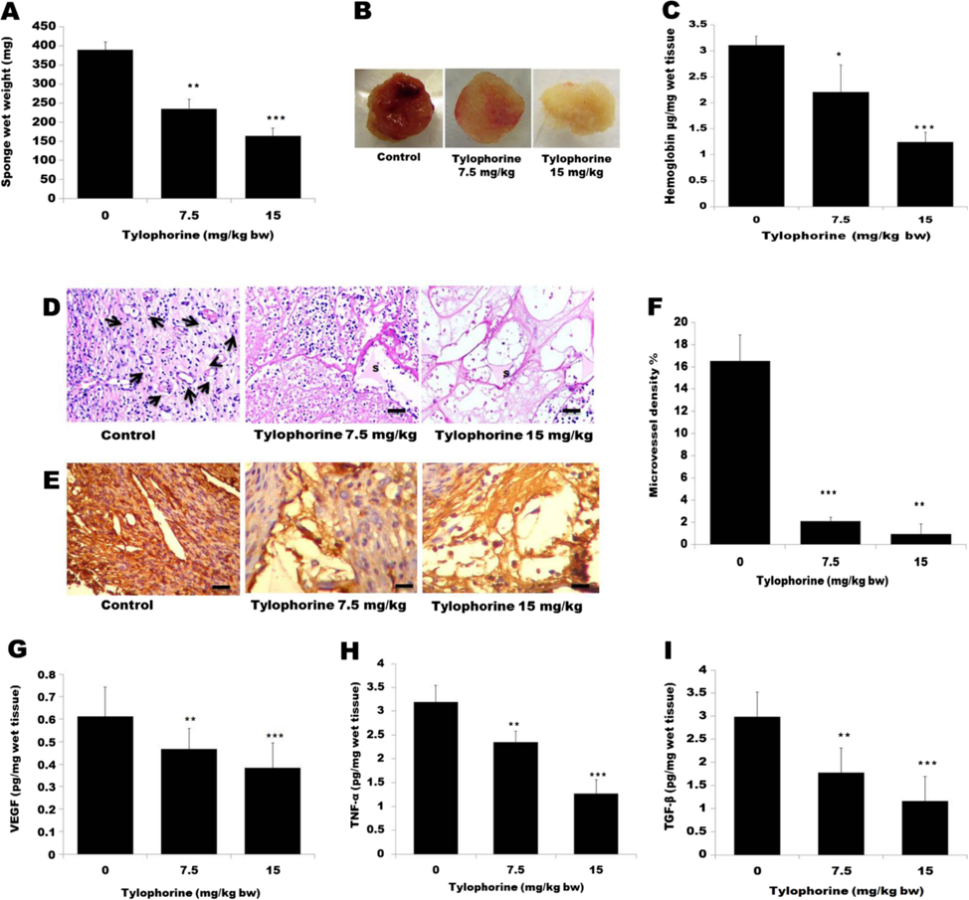
**Effect of tylophorine on sponge implant angiogenesis assay in vivo.** Sterile circular sponge discs were inserted subcutaneously into male Swiss albino mice and treated with tylophorine for 14 days. Mice were sacrificed and sponge was excised, **(A)** weighed and **(B)** photographed. Data were presented as means ± SEM, n = 10. **p < 0.01; *** p < 0.001 versus control group. **(C)** Excised sponge was homogenized in Drabkin Reagent to quantify the hemoglobin level. The content of hemoglobin in each implant is expressed as g/dl/per wet tissue. Data were presented as means ± SEM, n = 10. ***p < 0.001 versus control group. **(D)** Representative histological sections (5 μm, stained with H & E) of sponge implants. The pores of the sponge matrix are filled with inflammatory cells, spindle-shaped fibroblasts, blood vessels. In the control group fibrovascular tissue is denser and more vascularized compared with the tylophorine -treated groups at the doses of 7.5 and 15 mg/kg, Arrows shows the blood vessels in control group. s: sponge: Scale bar: 100 μm. **(E)** Immunohistochemical staining of sponge with CD31. Sections from the sponge tissue that were either untreated or -treated were incubated with anti-CD31 overnight at 4°C and stained with ABC-reagent according to the manufacturers protocol. Scale bar: 100 μm. **(F)** Effects of tylophorine on microvessel density (MVD) in sponge implants. Effect of tylophorine on cytokine levels **(G)** VEGF **(H)** TNF-α **(I)** TGF-β. Data were presented as means ± SEM, n = 10. **p < 0.01; ***p < 0.001 versus control group.

To determine whether tylophorine has an effect on angiogenesis *in vivo*, we performed a sponge implant angiogenesis assay in Swiss albino mice. Sponge disks were *s.c.* implanted into mice and treatment with tylophorine or DMSO was continued, once daily, for 14 days. Over 14 day experimental period, the weight of sponge granuloma tissues increased gradually in vehicle-control group, whereas in tylophorine treated group sponge weight was reduced dramatically (Figure 6A). Daily administration of tylophorine into the sponge implants caused a marked decrease in angiogenesis as evident by pictorial representation (Figure 6B) and decreased hemoglobin concentration (Figure 6C) in sponge granuloma tissues. In implants of control group, the hemoglobin levels were found to be 3.11 ± 0.17 μg Hb/mg wet tissue (n = 10); versus 2.21 ± 0.52 μg Hb/mg (tylophorine 7.5 mg/kg; n = 10) and 1.24 ± 0.19 μg Hb/mg wet tissue (tylophorine 15 mg/kg; n = 10). The difference in between control and treated groups were further confirmed by morphometric analysis of implants that the number of blood vessels was markedly lower in the treated groups as compared to control group (Figure 6D), which was confirmed by staining with CD31. It was observed that tylophorine treatment significantly reduced the CD 31 expression as compared to control group (Figure 6E). The microvessel density was statistically lowered in tylophorine treated sponge tissue (Figure 6F). Subsequently, it was sought to correlate this change in vascularization with change in the level of VEGF in the implants. It was found that tylophorine significantly inhibited VEGF level in sponge implant tissues (Figure 6G). The inflammatory components of the sponge-induced inflammation were determined by estimating the numbers of the leukocytes in the implant by assaying levels of pro-inflammatory cytokines TNF-α. Tylophorine at 15 mg/kg reduced the TNF-α level by 41.81% (Figure 6H). As shown in Figure 6I, there was a clear decrease in the TGF-β levels (38.92 and 59.73% at 7.5 and 15 mg/kg respectively) after tylophorine treatment.

### Tylophorine inhibited tumor growth i*n vivo*

**Figure 7:**
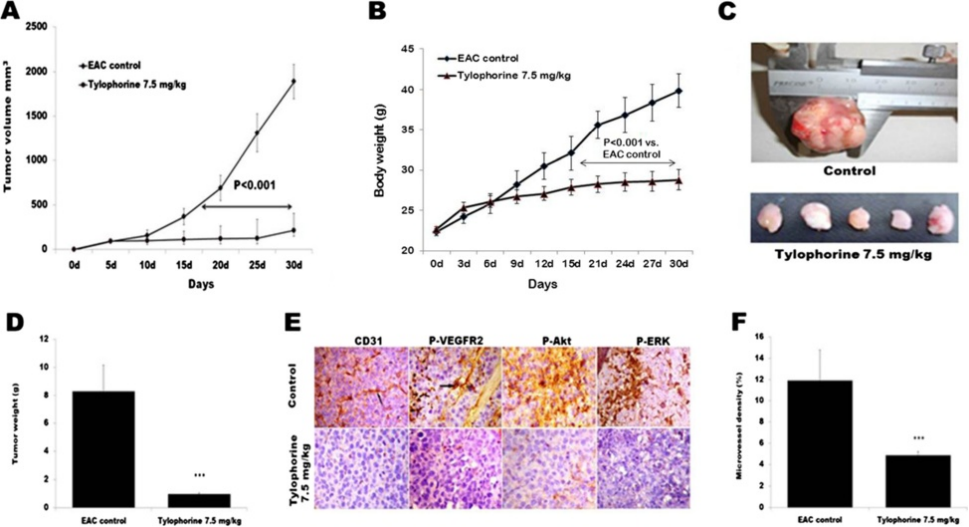
**Effect of tylophorine on tumor growth and VEGFR2 phosphorylation in EAC tumor model.** 15 × 10^6^ EAC cells/mouse were injected *s.c.* into 5–6 week old Swiss albino mice. After solid tumors grew to ~ 100 mm^3^, the mice were *i.p.* treated with tylophorine (7.5 mg/kg bw). Tumor growth was measured with calipers every fifth days using the formula: Tumor volume(mm^3^) = (width)^2^ × (length) × π/6. Tylophorine treatment decreases **(A)** tumor volume **(B)** body weight. Data were presented as means ± SEM, n = 15. **(C)** Representive images of solid tumor lump shows tylophorine treated group is significantly smaller than those in the control group. **(D)** The tumor tissue was removed from mice at 30 days after treatment and weighed. **(E)** Immunohistochemical staining of tumor tissue (n = 6) with antibodies against P-VEGFR2, P-ERK, P-Akt and CD31. **(F) **% MVD was determined by selecting the blood vessel (CD31) area per field in selected vascularized areas divided by the whole area. Data were presented as means ± SEM, n = 6. ***p < 0.001 versus control group.

Prompted by the *in vitro* and *in vivo* data supporting a potential antiangiogenic activity of tylophorine, we examined the *in vivo* efficacy of tylophorine on the growth of mouse Ehrlich ascites solid tumor, which is highly dependent on angiogenesis. As compared to control group treated with vehicle, tylophorine -treated group showed slower growth kinetics of EAC solid tumor (Figure 7A). It was found that treatment with tylophorine significantly led to suppression of EAC solid tumor volumes when compared with the control group. The average tumor volume in the control group increases from 91.35 ± 21.64 mm^3^ to 2139.05 ± 193.09 mm^3^ after 30 days, whereas the average tumor volume in the tylophorine-treated mice increased from 93.28 ± 31.98 mm^3^ to 213.96 ± 65.61 mm^3^ (Figure 7A). The body weights of animals corresponded well with the growth of tumors in respective group of animals (Figure 7B). The effect of tylophorine alone on body weight of normal mice is depicted in Additional file 2: Figure S2. Quantitatively weights of tumor lumps treated with tylophorine were also found smaller (p *<* 0.001) as compared to control group (Figure 7C). The average tumor weight in the control group was 8.34 ± 1.85 g; whereas the average tumor weight in the tylophorine -treated group was found to be 0.98 ± 0.07 g (Figure 7D) indicating that proliferation rate of tumor cells in mice was greatly inhibited by tylophorine. To further examine whether tylophorine could suppress tumor growth by inhibiting angiogenesis, tumor tissues were stained with specific antibodies against CD31, P-VEGFR2 (Tyr 1175), P-AKT, and P-Erk in Figure 7E. CD31 is a widely used endothelial marker for quantifying angiogenesis by calculating microvessel density (MVD) [42]. Our data showed that the average number of blood vessels in tylophorine treated group is 4.87 ± 0.34 blood vessels/HPF (Figure 7F) as compared with 11.93 ± 2.84 blood vessels/HPF in the control group (P < 0.001). Suppressed CD31 expression and decreased tumor volume and tumor weight suggests that tylophorine targets endothelial cells (ECs) as well as tumor cells. In addition, tylophorine down-regulated the expressions of P-VEGFR2, P-Akt, and P-Erk (Figure 7E) further demonstrating that tylophorine played an important role in suppressing angiogenesis at least partly through VEGFR2 signaling pathways.

### Tylophorine prolongs the survival of tumor bearing mice

**Figure 8:**
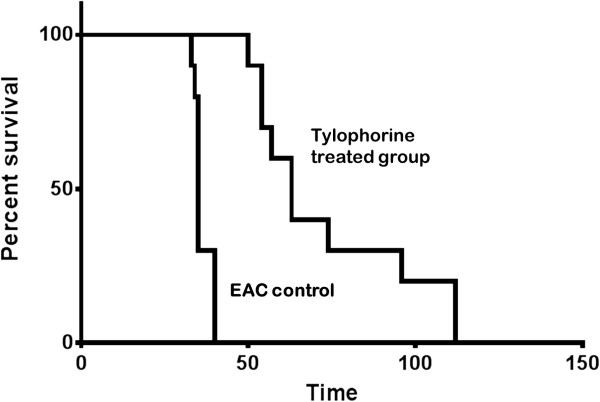
Kaplan-Meier survival curve for tylophorine treated EAC mice in comparison to EAC control group (n = 15).

The tumor bearing mice administered with DMSO or tylophorine (7.5 mg/kg bw) for 30 days were observed and the days of survival were recorded. With tylophorine treatment, the survival of tumor bearing mice significantly increased from 35.2 ± 1.29 days to 70.3 ± 3.28 days (Figure 8) as obtained by Kaplan Meier’s survival analysis (Figure 8).

### Tylophorine located at the ATP-binding sites of VEGFR2 kinase domain

**Figure 9:**
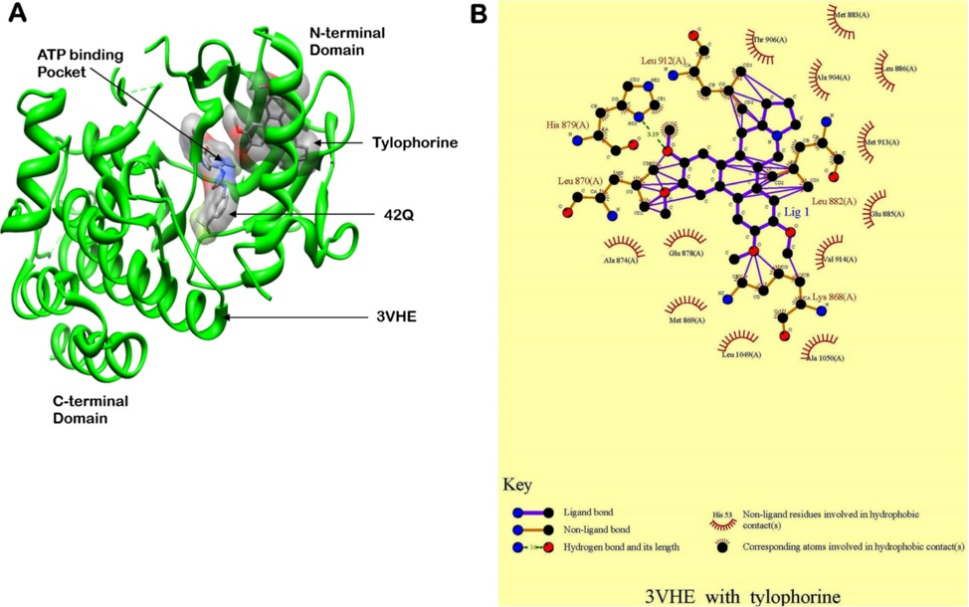
**Tylophorine interacted with the ATP-binding sites of VEGFR2 kinase domain. (A)** Binding sites of original crystallized bound 42Q and docked tylophorine ligands in volumetric structure in grey colors with VEGFR2 protein (PDB-ID: 3VHE in green ribbon structure) has been created by Chimera program. Both ligands are showing different binding site location. Different types of surface colors of both ligands are showing chemical nature of involved heteroatoms. 42Q ligand has Oxygen (in red colour), Nitrogen (in blue colour), carbon (in grey colour) and Fluorine (in green colour) while Tylophorine contains only Oxygen and carbon heteroatoms. **(B)** 2-dimensional interaction map of tylophorine and involved amino acids of 3VHE proteins were calculated by LigPlot Software. Key describes the types of involved interaction and bonds.

We next analyzed the binding pattern between tylophorine and VEGFR2 kinase domain to further understand how tylophorine exerted anti-angiogenesis effects via VEGFR2 and its signaling pathways. When molecular docking simulation between tylophorine ligand and VEGFR2 protein was analyzed, it was found that tylophorine has bound at slightly different location toward N-terminal domain from original bound ligand 42Q with-7.00 Kcal/mol binding affinity in the ATP binding pocket (Figure 9A). There are five amino acids *i.e.*, Lys868, Leu870, His879, Leu882 and Leu912 are actively involved in the binding of tylophorine. His879 is an active amino acid of the ATP binding pocket has participated in hydrogen bond with tylophorine. Rest amino acids are hydrophobic in nature and have made strong π-π bonds with the ligand. Therefore hydrophobic interaction is more dominant than hydrogen and electrostatic interaction in tylophorine-VEGFR2 complex (Figure 9B). When structure of tylophorine was inspected, it has found that its core structure has made up with three fused benzene rings which are also hydrophobic nature suggesting, it may be reason for dominancy of hydrophobic interaction. Such binding pattern of tylophorine within VEGFR2 may prohibit the binding of the ATP at its binding pocket and in this way it has provided a direction for development of small natural inhibitors.

## Discussion

The present study demonstrated that tylophorine exhibited anti-angiogenic activities *in vivo* and suppressed key steps involved in angiogenesis including proliferation (Figure 1C), migration (Figure 2A), invasion (Figure 2B), tubulogenesis (Figure 2C) and expression of pro-MMP2 (Figure 4B) as detected by gelatin zymography in endothelial cells. By directly blocking VEGFR2 phosphorylation and activation, tylophorine inhibited VEGFR2 kinase activity (Figure 3B) as well as suppressed VEGFR2 signaling pathway (Figure 4A) in vitro. Supporting evidences concerning in vivo anti-angiogenesis effects of tylophorine then came from sponge implant angiogenesis model and Ehrlich ascites carcinoma tumor model. Tylophorine significantly inhibited blood vessels formation in sponge implant assay (Figure 6) and significantly suppressed tumor growth accompanied by reduction in microvessel density (MVD) in tumor tissues (Figure 7).

Our study provides a novel and mechanistic insights into the mechanism by which tylophorine affects the multiple facets of vascular endothelial angiogenic signaling through VEGFR1 and VEGFR2. Phosphorylated Tyr1175 of VEGFR2 mediates activation of the mitogen-activated protein kinase/ERK cascade and was shown to contribute to cell proliferation in endothelial cells [38-40]. Src family kinase is substantially involved in VEGF-induced angiogenesis *in vitro* and *in vivo*[43-45]. Other signaling molecules that have been involved in VEGF-induced migration through VEGFR2 include FAK and its substrate paxillin, which are participated in focal adhesion during cell migration [6,46]. By interacting between FAK and Src, a dual kinase complex FAK-Src forms, and is activated by multiple integrin-regulated linkages [47]. Recent studies show that inhibition of ERK, phosphoinositide 3-kinase, PDT1/Akt and FAK downstream of VEGFR2 has emerged as a target for anticancer therapy [48]. AKT/mTOR/ribosomal protein S6 kinase (p70S6K) signaling has also been identified as a novel, functional mediator in angiogenesis [48,49]. VEGFR1 plays a positive role in promoting tumor angiogenesis by cross-talks among epithelial cells and other cell types because VEGFR1 is expressed not only endothelial cells but also on macrophage lineage cells and tumor epithelial cells [50]. VEGFR1 is a kinase-impaired RTK, and may signal in the context of a receptor heterodimer [51].

Our studies indicated that tylophorine interfered with the binding of VEGFR2 and reduced the autophosphorylation of VEGFR2 whereas; tylophorine did not affect the VEGF binding to VEGFR1. We also found that a half-maximum inhibitory concentration 9.2 μM (Figure 3B) of tylophorine significantly blocked the kinase activity of VEGFR2. Further it was observed that tylophorine modulates VEGF-mediated vascular permeability and angiogenesis by inhibiting phosphorylation of Akt, ERK, FAK, mTOR, Src and eNOS (Figure 3C) in endothelial cells *in vitro*. In addition, it was also found that tylophorine inhibited MMPs activity in a dose-dependent manner, suggesting that decreased MMPs activity might be also responsible for interfering with the binding of VEGF to VEGFR2, and thus inhibiting the neo-angiogenesis process [52]. Furthermore, ROS was reported as a downstream signaling of VEGFR2 and served as a survival mediator in supporting endothelial cell proliferation [53]. Our results demonstrated that the ROS level decreased significantly after tylophorine administration, which might be a consequence event of decreased VEGFR2 activity. All these results suggested that tylophorine inhibits the VEGFR2 signaling pathways.

As mentioned above, dimerization within the extracellular domain of VEGFR2 could induce the autophosphorylation on numerous tyrosine residues within its intracellular domain. The phosphorylation is an ATP consuming process. The ATP-binding region lies between N-terminal lobe and C-terminal lobe within VEGFR2 catalytic domain. Many kinase inhibitors could exert their inhibitory effects through purely or partially competing against the adenosine triphosphate (ATP) and subsequently suppressing the receptor autophosphorylation. They were acting as ATP minetics that bound to this site and competed with cellular ATP [33]. In this study, tylophorine could stably locate at the ATP-binding pocket near the hinge region. There are five amino acids i.e., Lys868, Leu870, His879, Leu882 and Leu912 at the ATP pocket were essential for the stable conformation of VEGFR2/tylophorine complex. Rest amino acids are hydrophobic in nature and have made strong π-π bonds with the ligand. All the unique binding modes largely promoted the conformational stability of the tylophorine/VEGFR2 complex.

## Conclusion

Overall our study indicated that tylophorine exerted potent anti-angiogenesis activities via specifically targeting VEGFR2 and its signaling pathway. As a natural inhibitor against VEGFR2, tylophorine is a promising candidate for development of anti-angiogenesis agents.

## Methods

### Chemicals and reagents

Tylophorine was purchased from Enzo Life Sciences (UK) Ltd. Phosphate-buffered saline (PBS), Tween 20, fetal bovine serum (FBS), bovine serum albumin (BSA), phenylmethanesulfonyl fluoride (PMSF), ethylenediaminetetraacetic acid (EDTA), heparin, HEPES buffer, penicillin, streptomycin, NaHCO_3_, amphotericin B, dimethyl sulfoxide (DMSO) and gelatin were obtained from Sigma (St. Louis, MO, USA). Tylophorine was dissolved in 0.1% DMSO to form a 100 mM solution, stored at-20°C in small aliquots until needed and protected from light, and then diluted to various concentrations as needed. Growth factor-reduced Matrigel was purchased from BD Biosciences (San Diego, CA). The antibodies anti-β-actin, anti-VEGFR2, anti-Src, anti-FAK, anti-ERK1/2, anti-AKT, anti-mTOR, anti-CD31, phospho-specific anti-VEGFR2 (Tyr^1175^), anti-c-Src (Tyr^416^), anti-FAK (Tyr^576/577^), anti-ERK1/2 (Thr^202^/Tyr^204^), anti-AKT (Ser^473^), anti-mTOR (Ser^2448^), Phototope® HRP Western blotting detection System (LumiGLO® chemiluminescent reagent and peroxide), TMB substrate and stop solution were delivered from Cell Signaling Technology (Danvers, MA, USA). VEGF, IL-6, IL-8, TNF-α, and IFN-γ were procured from R and D systems (MN, USA). M199 medium and sodium dodecyl sulfate polyacrylamide electrophoresis (SDS-PAGE) gels were acquired from Invitrogen (Life Technologies, Grand Island, NY, USA).

### Cell lines and cell culture

Human umbilical vascular endothelial cells (HUVECs) (Clonetics, Lonza, Basel, Switzerland) were cultured in endothelial cell growth medium (ECGM): M199 medium supplemented with 20% FBS, 20 μM bECGF, 0.1 mg/mL heparin, 15 mM HEPES buffer, 50 IU/L penicillin, 50 mg/L streptomycin, 44 mM NaHCO_3_, and 50 μg/mL amphotericin B under a humidified chamber at 37°C with 5% CO_2_.

### Cell viability assay

HUVECs (5 × 10^4^ cells/well) were plated onto a gelatinized 24-well culture plate and cultured in ECGM containing 20% FBS. HUVECs were treated with DMSO (0.1%) or different concentrations of tylophorine (0, 2.5, 5, 10, 20 μM) for 24, 48 and 72 h. Cell viability was determined by MTT assay as described previously [54]. After 4 h of incubation, the absorbance was measured at 450 nm with a microplate reader (Biorad, USA). The results were calculated from six replicates of each experiment. Three independent experiments were performed.

Next, we determined the effects of tylophorine on VEGF-induced cell viability. HUVECs (5 × 10^4^ cells/well) were starved with ECGM containing 0.5% FBS for 24 h. After the pre-incubation, cells were treated with or without VEGF (10 ng/mL) and DMSO (0.1%) or different concentrations of tylophorine and incubated for another 24 and 48 h. Cell viability was quantified by MTT assay. The group without VEGF and tylophorine treatment was set as 100%. The results were the means calculated from six replicates of each experiment. Three independent experiments were performed.

### BrdU incorporation assay

DNA synthesis was determined by bromodeoxyuridine(BrdU) labeling assay using Cell Proliferation ELISA, BrdU (colorimetric) kit. In brief, 5 × 10^4^ HUVECs per well (100 μl in ECGM + 20% FBS) were seeded in a gelatin coated for overnight attachment. Then the cultivated medium was replaced with serum-free medium supplemented with 10 ng/mL VEGF as well as different concentrations of tylophorine in a final volume of 100 μl /well. After 24 h, cells were labeled with BrdU (2 h, 37°C, 4 h), incubated with Fix Denat solution (30 min, 20°C), and reincubated with Anti-BrdU POD (90 min, 20°C). The absorbance was read at 450 nm in a microplate reader (Biorad, USA). The assay was repeated three times independently.

### Lactate dehydrogenase (LDH) toxicity assay

The LDH release assay was performed using a cytotoxicity detection kit plus (LDH) (Roche Diagnostics) according to the manufacturer’s instructions. In brief, HUVECs were seeded in 96-well plate at a density of 5 × 10^4^ cells per well. After incubation with various concentrations of tylophorine for 24 h, cell supernatants were collected and analyzed. The absorbance of formed formazan was read at 490 nm on a microplate reader (Biorad, USA). The LDH content of each sample was calculated according to the following formula: Cytotoxicity(*%*) = [(experimental value − low control)/(high control − low control)] × 100. The assay was repeated three times independently.

### Endothelial cell migration assay: wound healing

HUVECs (5 × 10^4^ cells/well) were allowed to grow to full confluence in 6-well plates pre-coated with 0.1% gelatin and then starved with ECGM containing 0.5% FBS for 6 h to inactivate cell proliferation. After that, cells were wounded with pipette tips and washed with PBS. ECGM supplemented with 0.5% FBS was added into the wells with or without VEGF (10 ng/mL) and DMSO (0.1%) or different concentration of tylophorine. Images of cells were taken using an inverted microscope (Eclipse TS100, Nikon, Japan) at 100 × magnification after 16 h of incubation in a humidified atmosphere with 5% CO_2_ at 37°C. The migrated cells were observed from three randomly selected fields and quantified by manual counting. Cells receiving only DMSO served as a vehicle control. Inhibition percentage was expressed as percentage of the vehicle control (100%). The assay was repeated three times independently.

### Endothelial cell invasion assay

Cell invasion assay was performed using Transwell chambers with 6.5 mm diameter polycarbonate membrane (8-μm-sized pores). Upper surfaces of transwell inserts were coated with matrigel. The bottom chamber of the apparatus contained 600 μL of endothelial cell medium supplemented with 10 ng/mL VEGF or tylophorine at different concentrations. The HUVECs (100 μL) were added to the upper chamber (5 × 10^4^ cells/well) and incubated in endothelial cell medium. After 24 h incubation at 37°C, non-invasive cells on the upper membrane surfaces were removed by wiping with cotton swabs. Cell invasion was quantified by counting cells on the lower surface using phase contrast microscope (Eclipse TS100, Nikon, Japan) at 100 × magnification. The results were the means calculated from three replicates of each experiment. The assay was repeated three times independently.

### Endothelial cell capillary-like tube formation assay

Matrigel™ basement membrane matrix (growth factor reduced) (BD Biosciences, San Jose, CA, USA) was thawed at 4°C, pipetted into pre-chilled 24-well plates (100 μL matrigel/well) and incubated at 37°C for 45 min. HUVECs were firstly incubated in ECGM supplemented with 0.5% FBS for 10 h and then treated with DMSO (0.1%) or different concentrations of tylophorine for 30 min before seeding. Cells were collected and placed onto the layer of matrigel (5 × 10^4^ cells/well) in 1 mL of ECGM supplemented with 0.5% FBS, followed by the addition of VEGF (10 ng/mL). After 24 h of incubation with 5% CO_2_ at 37°C, the network-like structures of endothelial cells were examined under an inverted microscope (Eclipse TS100, Nikon, Japan) at 100 × magnifications. Branching points in three random fields per well was quantified by manual counting. Cells receiving only DMSO (0.1%) served as a vehicle control. Inhibition percentage was expressed as percentage of the vehicle control (100%). The assay was repeated three times independently.

### VEGFR binding assay

VEGFR binding assay was performed as described previously [55]. Briefly, VEGF (50 ng/mL) in 50 μL of PBS were immobilized to 96-well plates. The wells were washed and blocked with 3% bovine serum albumin (BSA) in PBS for 2 h. Tylophorine with 1% BSA in PBS were added with VEGFR1 (Flt-/Fc, 20 ng/mL; R&D Systems, Minneapolis, MN) or VEGFR2 (KDR/Flk-1; 20 ng/mL; R&D Systems, Minneapolis, MN) to VEGF-coated wells. After 3 h incubation, the wells were washed thrice with PBST. Flt-1 or KDR/Flk-1 bound to VEGF was determined by biotinylated anti-human IgG (Dako) and horseradish peroxidase–conjugated streptavidin (Sigma), developed with tetramethylbenzidine substrate reagent (BD Biosciences), and quantified by measuring the absorbance at 450 nm.

### In vitro VEGFR2 kinase inhibition assay

*In vitro* VEGFR2 tyrosine kinase activity was assayed using HTScan® VEGFR2 kinase assay kit (Cell Signaling Technology, USA) combined with colorimetric ELISA detection as described previously [56]. The final reaction system included 60 mmol/L HEPES (pH 7.5), 5 mmol/L MgCl_2_, 5 mmol/L MnCl_2_, 3 μmol/L Na_3_VO_4_, 1.25 mmol/L DTT, 20 μmol/L ATP, 1.5 μmol/L substrate peptide, 100 ng of VEGF receptor kinase and indicated concentrations of tylophorine. The results were expressed as percent kinase activity of the vehicle control (100%), and IC_50_ was defined as the compound concentration that resulted in 50% inhibition of enzyme activity. The kinase assay was performed thrice independently.

### Western blotting analysis

In brief, cell lysates (50 μg) were separated by 8% SDS PAGE and transferred to polyvinylidene difluoride membranes. Membranes were then incubated with primary antibodies including phosphorylated and/or total VEGFR2, ERK1/2, AKT, mTOR, c-Src, FAK, eNOS and β-actin (Cell Signaling Technology, USA). After overnight incubation at 4°C, membranes were washed with TBST three times and then incubated with secondary antibodies at room temperature for 2 h. Immunoreactive bands were then visualized by the enhanced chemiluminescence (ECL) detection system (GE healthcare). Cells receiving only DMSO (0.1%) served as a vehicle control. Three independent experiments were performed in triplicates.

### Gelatin zymography

HUVECs (80% confluent) were washed with serum-free M199 and incubated with or without VEGF (10 ng/mL) containing tylophorine for 20 h. The proteins in conditioned medium were size fractionated on a 10% SDS-polyacrylamide gel impregnated with 0.1% gelatin. MMP2 and other MMPs were activated in gel for 18 h at 37°C. Gels were fixed, stained with 0.25% Coomassie brilliant blue R250, and destained. Gelatinase activity was visualized as cleared band on the stained gel.

### Measurement of reactive oxygen species

2’7’-Dichlorofluorescein diacetate (DCFH-DA, Sigma, St. Louis, MO) was used to measure ROS formation. After exposed to different concentrations of tylophorine for 24 h, endothelial cells were then incubated in 10 μM DCFH-DA at 37°C for 20 min. Cells were washed with PBS three times to remove DCFH-DA that not entered in cells. The fluorescence of oxidized probe was measured using a microplate plate reader (Synergy Mx Multimode, Biotek, US). The fluorescence was visualized immediately at wave lengths of 485 nm for excitation and 530 nm for emission by inverted fluorescence microscope. Total green fluorescence intensities of each well were quantified using image analysis software.

### Cytokine immunoassays

Secreted IL-6, IL-8, TNF-α, IFN-γ and MMP-2 levels in tylophorine treated HUVEC culture medium were measured using an ELISA kit (R and D Systems, MN, USA) according to manufacturer’s instructions.

### Nitric oxide (NO) measurement

Secreted NO level in tylophorine treated HUVEC culture medium were measured using a Nitric oxide colorimetric assay kit (Biovision, USA) according to manufacturer’s instructions.

### Sponge implant angiogenesis assay

Sponge implant assay was performed as described previously [57,58]. Sterile circular sponge discs were inserted subcutaneously into male Swiss albino mice (n = 10). The day of sponge insertion was taken as day 0. Commencing day 1, animals were treated with tylophorine (7.5 and 15 mg/kg bw) from day 1 to day 14. On the day following the last injection (day 15) mice were sacrificed and the sponges were excised, weighed and photographed. Sponges were bisected; one half was fixed in 10% formalin and embedded in paraffin wax. Sections (5 μm) were stained with hematoxylin/eosin for identification of blood vessels. The second half of the sponge was weighed, homogenized in 2 mL of sterile PBS at 4°C, and centrifuged (2000 × *g* for 30 min) to quantify level of VEGF, TNF-α and TGF-β. The extent of the vascularization of the sponge implants was assessed by the amount of hemoglobin (Hb) detected in the tissue using the Drabkin method [59]. All procedures for animal experimentation used were approved by the Institutional Animal Ethics Committee.

### In vivo antitumor activity

Ehrlich ascites carcinoma (EAC) cells (15 × 10^6^) were implanted subcutaneously into female Swiss albino mice, 5-6 weeks old, weighing 20-25 g [60,61]. After tumors became palpable, the mice were divided into two groups (n = 15 each) based on the tumor size of each mouse so that the average tumor volume was equal between the groups. One group of mice (n = 15) was injected with vehicle (0.1% DMSO) and the other group (n = 15) were injected with 7.5 mg/kg bw tylophorine, intraperitoneally (*i.p.*), every day. The tumor volume was measured using a vernier caliper and calculated according to the modified ellipsoid formula: Tumor volume(mm^3^) = (width)^2^ × (length) × π/6. The effect of tylophorine on percentage increase in life span was calculated on the basis of mortality of the experimental mice (n = 15) in solid tumor [33]. For determination of mean survival time (MST) and percentage increased life span (% ILS), animals were allowed to natural death [38]. After 30 days of treatment, the mice were sacrificed and whole tumor tissues were excised, weighed and photographed. Excised tumors were fixed in 10% formaldehyde and embedded in paraffin. 5-μm sections were stained with hematoxylin and eosin (H & E) and immunostained with antibodies against mouse CD31 (Santa Cruz biotechnology, UK), VEGF, P-ERK, P-Akt, and P-VEGFR2 (Cell Signaling, USA), and visualized by appropriate biotin-conjugated secondary antibodies followed by immmunoperoxidase detection with the Vectastain ABC Elite kit (Linaris, Germany) and diamino-benzidine (DAB) substrate (Vector, UK). Counterstaining was performed with hematoxylin. Microvessel density was calculated using Image J software (NIH, Bethesda, MD) [28,33]. All procedures for animal experimentation used were approved by the Institutional Animal Ethics Committee.

### Molecular docking

Computational based study of molecular interaction between tylophorine and VEGFR2 receptor was carried out using Autodock Vina software [62]. Ligand structures were optimized by using MarvinScketch program. Protein and ligand were prepared for docking simulation by adding of Gasteiger partial charges [63] and polar hydrogen with the help of AutoDock Tool program. X-ray crystal structures of VEGFR2 protein (PDB-ID: 3VHE) with small molecule, 42Q was downloaded from Protein Data Bank (http://www.rcsb.org). Water molecules and other heteroatom were manually removed out from the protein structures. 3D structure of tylophorine ligand was downloaded from PubChem database (http://www.ncbi.nlm.nih.gov/PubChem). A grid cube box with 60Åx60Åx60Å dimension was centered on the originally crystallized 42Q ligand for searching the most suitable binding site of tylophorine during molecular docking simulation and exhaustiveness option was set up at 8. Chimera (http://www.cgl.ucsf.edu/chimera) and LigPlot [64] programs were used to analyze and visualizing the molecular interaction between the ligand and receptor with default parameter.

### Statistical analysis

The data were analyzed using SigmaStat 3.5 software. Results were presented as the mean ± S.E. from at least three independent experiments. One-way analysis of variance (ANOVA) was followed by the Newman-Keuls test, when appropriate, to determine the statistical significance of the difference between means. The Mann–Whitney *U* test was used to compare microvessel density in different tumor samples. A *p* value of < 0.05 was considered statistically significant.

## Competing interest

The authors’ declared that they have no competing interest.

## Authors’ contributions

SS designed study; acquired, analyzed and interpreted data; performed statistical analysis and drafted and revised the manuscript. PKK participated in acquisition of data, statistical analysis and manuscript preparation. SK, RK and AAA participated in experimental data acquisition and revision of manuscript. All authors read and approved the final version of manuscript.

## Supplementary Material

Additional file 1: Figure S1 Effect of tylophorine on growth inhibition in HUVECs. Click here for file

Additional file 2: Figure S2 Effect of tylophorine on body weight in normal mice treated with tylophorine at 7.5 mg/kg. Click here for file
