# Supplementary figures and images for: Tylophorine, a phenanthraindolizidine alkaloid isolated from Tylophora indica exerts antiangiogenic and antitumor activity by targeting vascular endothelial growth factor receptor 2–mediated angiogenesis
Source: Mol Cancer. 2013 Jul 29;12:82. doi: 10.1186/1476-4598-12-82 (PMC3733984; doi:10.1186/1476-4598-12-82)

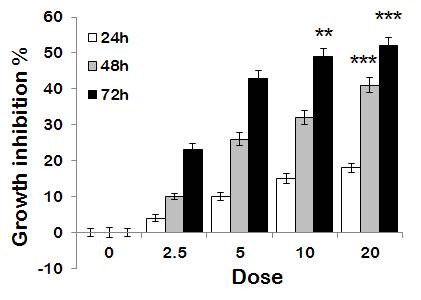

Supplement: Additional file 1: Figure S1 — Effect of tylophorine on growth inhibition in HUVECs. [file 1476-4598-12-82-S1.jpeg]

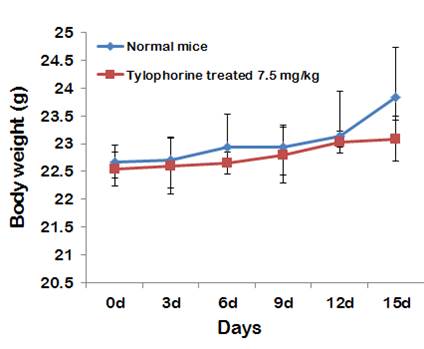

Supplement: Additional file 2: Figure S2 — Effect of tylophorine on body weight in normal mice treated with tylophorine at 7.5 mg/kg. [file 1476-4598-12-82-S2.jpeg]
